# Uncovering COVID-19 infection determinants in Portugal: towards an evidence-based spatial susceptibility index to support epidemiological containment policies

**DOI:** 10.1186/s12942-023-00329-4

**Published:** 2023-04-06

**Authors:** André Alves, Nuno Marques da Costa, Paulo Morgado, Eduarda Marques da Costa

**Affiliations:** 1 Centre of Geographical Studies, Institute of Geography and Spatial Planning, University of Lisbon, 1600-276, Lisbon, Portugal; 2 Associate Laboratory TERRA, 1349-017 Lisbon, Portugal

**Keywords:** COVID-19, Health determinants, GIS, Multicriteria decision analysis, Non-pharmacological interventions, Spatial-based policies, Spatiotemporal analysis

## Abstract

**Background:**

COVID-19 caused the largest pandemic of the twenty-first century forcing the adoption of containment policies all over the world. Many studies on COVID-19 health determinants have been conducted, mainly using multivariate methods and geographic information systems (GIS), but few attempted to demonstrate how knowing social, economic, mobility, behavioural, and other spatial determinants and their effects can help to contain the disease. For example, in mainland Portugal, non-pharmacological interventions (NPI) were primarily dependent on epidemiological indicators and ignored the spatial variation of susceptibility to infection.

**Methods:**

We present a data-driven GIS-multicriteria analysis to derive a spatial-based susceptibility index to COVID-19 infection in Portugal. The cumulative incidence over 14 days was used in a stepwise multiple linear regression as the target variable along potential determinants at the municipal scale. To infer the existence of thresholds in the relationships between determinants and incidence the most relevant factors were examined using a bivariate Bayesian change point analysis. The susceptibility index was mapped based on these thresholds using a weighted linear combination.

**Results:**

Regression results support that COVID-19 spread in mainland Portugal had strong associations with factors related to socio-territorial specificities, namely sociodemographic, economic and mobility. Change point analysis revealed evidence of nonlinearity, and the susceptibility classes reflect spatial dependency. The spatial index of susceptibility to infection explains with accuracy previous and posterior infections. Assessing the NPI levels in relation to the susceptibility map points towards a disagreement between the severity of restrictions and the actual propensity for transmission, highlighting the need for more tailored interventions.

**Conclusions:**

This article argues that NPI to contain COVID-19 spread should consider the spatial variation of the susceptibility to infection. The findings highlight the importance of customising interventions to specific geographical contexts due to the uneven distribution of COVID-19 infection determinants. The methodology has the potential for replication at other geographical scales and regions to better understand the role of health determinants in explaining spatiotemporal patterns of diseases and promoting evidence-based public health policies.

## Introduction

The severe acute respiratory syndrome coronavirus 2 (SARS-CoV-2), responsible for the new coronavirus disease (COVID-19), has caused the biggest pandemic of the twenty-first century. Although the mortality rate is considerably lower in comparison to previous coronavirus epidemics, COVID-19 has a higher transmission rate [1] that forced the adoption of restrictive measures to contain human-to-human transmission, known as non-pharmacological interventions (NPI) [2].

The distribution of confirmed cases of COVID-19 had an uneven spread because the incidence of new infections was characterized by spatiotemporal heterogeneity at multiple scales [3]. The spatial patterns can be explained by multiple factors [4–10] that justify spatial variations in contagion exposure, vulnerability and susceptibility [11–18]. Different NPI management strategies, ranging from case isolation to comprehensive measures, are also explanatory of COVID-19 spatiotemporal variability [10, 19, 20]. Furthermore, the literature highlights the importance of spatial dependence stemming from geographical properties, such as proximity and contiguity to more-prone outbreak areas [21, 22].

Although there is already considerable literature devoted to identifying the determinants of COVID-19 infection and their effect on spatial patterns, with high methodological diversity [23], many of these studies do not summarize their evidence in a way that can be useful and integrated with public health measures for pandemic control. As stated by van Schalkwyk and McKee [24] there have been “challenges of translating knowledge into policy”. In public health and disease prevention, the use of spatial models tends to increase, with the growing availability and accessibility of data on disease incidence with higher granularity, leveraged by the need for heterogeneous territorially based public health policies [25, 26]. Therefore, in the current pandemic context is of the utmost importance the implementation of spatiotemporal surveillance systems that prioritize interventions in areas of higher infection risk [27] and a better incorporation of social factors into COVID-19 models can improve predictive accuracy for more tailored and effective responses [28]. Due to the uneven distribution associated with exposure to SARS-CoV-2 a spatial dimension is crucial [29]. In this perspective, estimating the spatial susceptibility and vulnerability in health-related subjects is essential to prevent disease spread [30–32] since knowledge of the distribution of susceptible individuals allows for the assessment of multiple  susceptibility levels [33].

In Epidemiology, susceptibility (to a disease) is understood as “the dynamic state of being more likely or liable to be harmed by a health determinant” [34]. Nevertheless, it is often used as a synonym for vulnerability, although the latter incorporates, beyond the position of relative disadvantage understood as the propensity to be adversely affected, the capacity for adaptation and resilience [34, 35]. Literature about the study of the unequal spatial propensity to COVID-19 infection can be found using both terms for the same type of analysis. In this paper, susceptibility was conceptualized in line with the definition of Porta [34]. From a methodological perspective our approach measures the relationship between the confirmed cases of the disease and the effect of indicators—e.g., determinants—in explaining the incidence patterns. This type of analysis is not only informative for public health policies targeted to different population groups [36, 37] but also essential in epidemic contexts to manage early warning systems [38, 39]. The classification of territorial units by their propensity to infection can be used for equity in pandemic and public health policies avoiding one-size-fits-all containment measures in favour of geographically-tailored interventions in areas more prone to diffusion [40, 41]. In this respect, spatial analysis and GIS have proved to be essential [23, 42].

In the case of Portugal, evidence-based knowledge about the existence of geographical contexts that are more favourable to transmission and outbreaks has been shown and highlighted by several authors [14, 43–45]. The spread of COVID-19 in the country has been associated with settlement patterns, transport networks, mobility behaviours, employment and other economic and social characteristics [8, 46–48]. However, indicators regarding the causes of the spread of the disease have not been properly integrated to serve as policy guidance in assisting public health decision-makers. Therefore, NPI management in Portugal has resulted exclusively from epidemiological indicators, ignoring social, economic and mobility information useful in differentiating local strategies. This comes of relevance because the inclusion of auxiliary information is crucial to model the disease [28] and identifying viral hotspots where lockdowns are most effective, or less transmission-prone areas where NPIs can be eased [49]. Similarly, spatial analysis can help analyze policy effects on transmission spatial dynamics. For the portuguese territory, Sá Marques et al. [14] suggested the need for territorial customized NPI, proposing a geographic mosaic based on a vulnerability risk index, while Pereira et al*.* [45] developed a risk conceptual model to monitor COVID-19 spatiotemporal dynamics.

This work explores the hypothesis of developing a municipal index of susceptibility to COVID-19 infection, for mainland Portugal, to serve as a basis for the adoption of NPI tailored to territorial specificities. The aims of this article are threefold: (i) identify significant determinants of COVID-19 infection for the first year of the disease; (ii) derive an infection susceptibility index that classifies municipalities from thresholds; (iii) assess the relationship between the susceptibility index and the incidence rate per population for tailored NPI. Overall, we deliver a proposal for a NPI spatial-based modelling framework, based on infection susceptibility and epidemiological data, to assist policy design and decision-making.

The study area is mainland Portugal at municipal scale. Worth to say, that the municipality (278 units) is the most disaggregated spatial unit with official data on COVID-19. As a southern European country, the continental territory had about 9.8 million inhabitants in 2021, seemingly peripheral to Europe but in a hub position between continents, it is an interesting case study because of the very disparate evolution of the number of cases and the spatial diffusion patterns. While in the first waves the timely containment ensured low incidence and low mortality, unlike in nearby countries such as Spain and Italy [50], in later periods the ineffectiveness of containment policies led to it becoming the country in the world with the highest COVID-19 incidence per inhabitant.

Despite vaccination campaigns, NPI remain important to contain SARS-CoV-2 outbreaks [51–53]. NPI have been adopted throughout the world to contain COVID-19 transmission and strategies varied [2, 54]. Considering as extremes the “China COVID zero policy” [20] on one hand and the *laissez-faire* Sweden approach [55] on the other, containment policies in Portugal can be considered as an intermediate approach, in balancing public health and economy. NPI were managed based on epidemiological monitoring but followed unclear criteria with contradictory decisions and lack of rationality during the first months, with a quasi-national scope as a “one size fits all”. After November 2020, a new paradigm began with measures depending on a risk threshold classification by the Directorate-General of Health (DGS), that categorized municipalities from the 14 day-cases per 100,000 inhabitants to define NPI at the municipal scale (Council of Ministers Resolution no 92-A/2020, November 2). Each category was associated with a set of NPI with harshness proportional to incidence. This risk classification consisted exclusively of the disease incidence, ignoring mortality and hospitalizations, and did not effectively represent the epidemiological risk, that is, “the probability of an adverse or beneficial event in a defined population over a specified time interval” [34].

Even though this later approach relied on known formal criteria and was spatial-based, it did not fit municipalities with small populations. For comparison purposes (Table 1) in Manteigas—a rural municipality with less than 3000 inhabitants—seven new cases were enough to exceed the first risk threshold, even though the contact tracing and isolation were simple. On the opposite way, the city of Lisbon—the capital of Portugal with more than 500 thousand inhabitants—could have more than 1300 cases, which is already community transmission, and not yet surpass the first risk threshold. Therefore, the lack of adequacy of this approach undermined timely containment in some cases while in others was excessively harsh. Following subsequent readjustments, the risk thresholds for low-density municipalities were changed. However, despite this improvement, some municipalities remained to be subject to a criterion with an excessively high value, resulting in challenges to containment. This way, the portuguese NPI approach was characterized by the late implementation of measures, particularly in more populated municipalities, in a reactive rather than preventive way, and although it was spatially based, it ignored the spatial variation of susceptibility.

**Table 1:** Comparison of risk thresholds and population numbers across municipalities

Municipality	Population (2021)	Number of 14-day cases to exceed the first risk threshold (after readjustment)
Lisbon	545,923	1311
Coimbra	140,838	339
Manteigas	2909	7 (14)

## Methodological steps

**Fig. 1:**
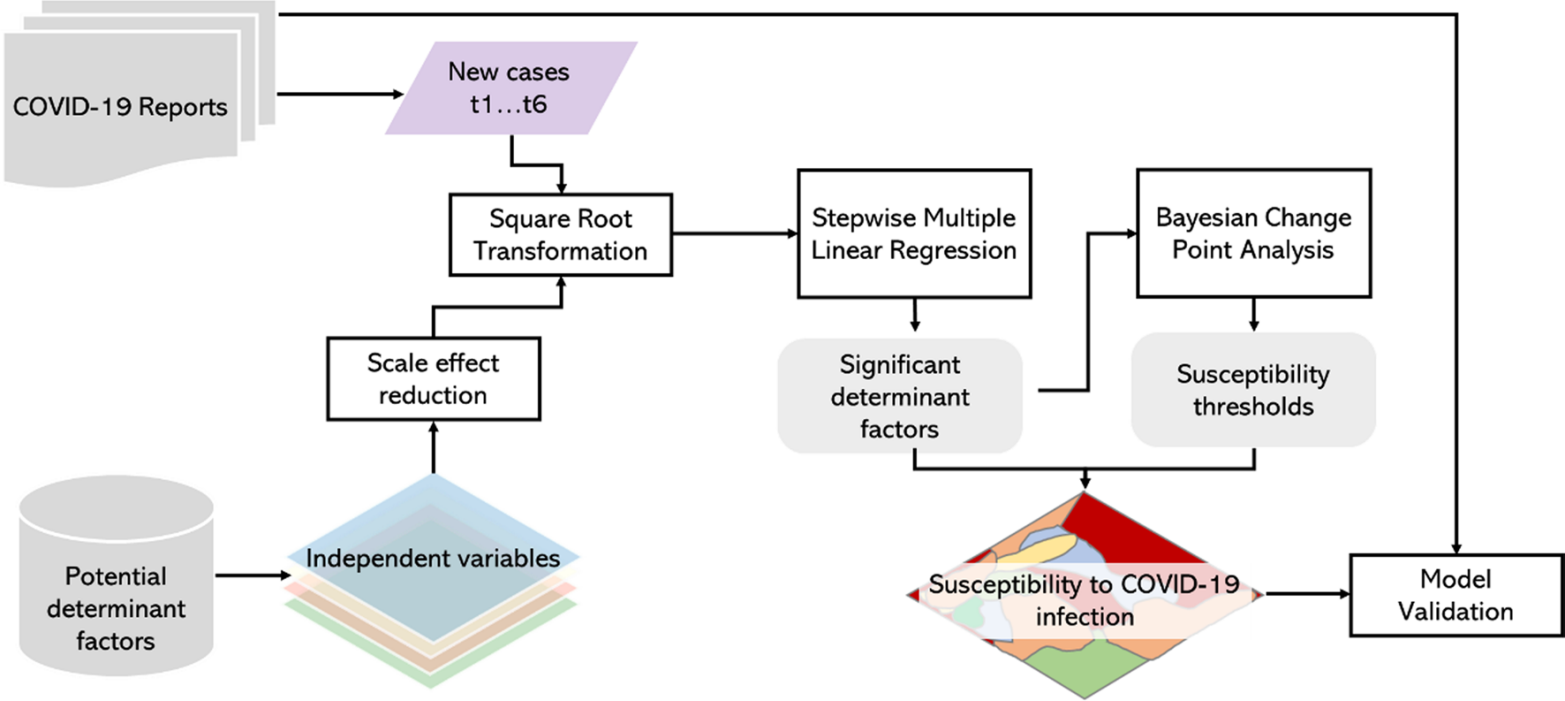
Methodological framework

The methodology applied to answer the objectives followed several steps (Fig. 1). Briefly, a multiple linear regression (MLR) was performed to reduce the dimensionality of a set of potential determinants of COVID-19 infection and obtain their influences in explaining patterns. Thereafter a Bayesian change point analysis (CPA) was applied to detect thresholds as changing points in the relationships between COVID-19 incidence and the most relevant factors, allowing the classification of municipalities accordingly to the susceptibility associated with each determinant. Afterwards, a weighted linear combination ensured the conjugation into a composite susceptibility index. The DGS risk levels and the susceptibility index were compared using the Spearman and contingency coefficients.

Given the need to uncover explanatory variables for COVID-19 spatiotemporal patterns, we supported the analysis using regression. Linear, generalized, mixed multi-level, non-linear and geographically based methods have been used for regression analysis to understand COVID-19 spatial dynamics and establish relationships with factors [5, 6, 8, 9, 46, 56, 57]. The choice of linear regression over more tuned methods is essentially due to two reasons. First, Thurner et al. [58] revealed that at most periods the COVID-19 infection curves of various countries entered linear growth phases, due partly to the effect of containment measures. Second, linearity tends to be lost (resulting in the famous S-curve) only when working with accumulated data, which was not the case because the periods modelled in this work corresponded to accumulations of 14 days which are relatively short and can be accommodated by a linear curve.

Regarding the use of a method for detecting change points, it has long been recognized that thresholds play a crucial role in understanding the spread of infectious diseases [59, 60]. This type of technique has precedents in COVID-19 modelling [61], however we are unaware of studies that rely on it to derive information for a susceptibility index.

### Data acquisition and treatment

The relationship between COVID-19 cases and their spatial determinants was performed in an aggregated data structure, i.e., an ecological analysis, whose explanatory variables were selected based on a literature review on the determinants of COVID-19 infection. These potential factors, ranging from indicators of urban density, employment by sector, to commuting patterns, were grouped into dimensions. Environmental and climatic data, used in some studies [7, 46] were not considered because defining a value that reflects the municipality’s reality would always revolve around simplification and bias. Furthermore, there is no consensus on the significance of these variables as predictors, resulting in conflicting findings in the literature, and normally less relevant than socioeconomic determinants [62].

**Fig. 2:**
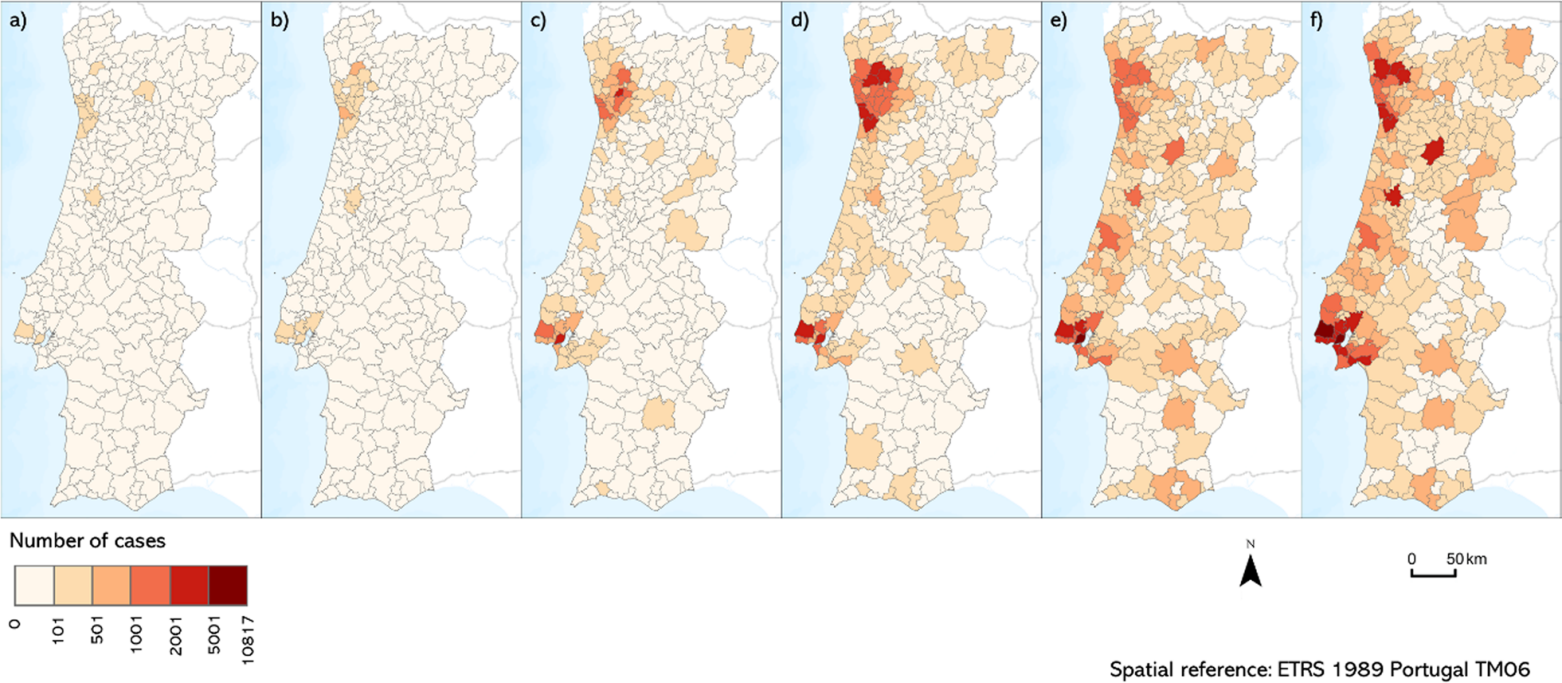
14-day cumulative incidence (dependent variables): **a** 1st wave start; **b** 1st wave peak; **c** 2nd wave start; **d** 2nd wave peak; **e** 3rd wave start; **f** 3rd wave peak

The data used has multiple sources. A total of 51 potential determinant factors (Table 2) were considered from Statistics Portugal (https://www.ine.pt/) and Social Chart (https://www.cartasocial.pt/). The epidemiological information (number of cases) was obtained from the COVID-19 situation reports of DGS [63] for 6 periods. The periods under analysis correspond to 14-day blocks of new cases of the disease, representative of the beginning and the peak of the first three waves of COVID-19 in Portugal between March 2020 and March 2021 (Fig. 2).

**Table 2:** Considered potential spatial determinants of COVID-19 cases. Source: Statistics Portugal and Carta Social

Dimension	Example of indicator(s)	Number
Age dimension	• Proportion of population by age group	4
Sociodemographic	• Population density, urbanization rate and average household size • Students enrolled by year of schooling • Beneficiaries of social and unemployment benefits • Public housing, average age of buildings and decayed dwellings	18
Mobility	• Use of public transport and personal vehicle in daily commute • Time duration of daily commuting route • Intermunicipal and interparish commuting	6
Economic	• Employment location quotients (LQ) for 16 sectors • Declared income, export value and gross value added of companies • Tourism overnight stays • Housing expenses and owner occupied housing ratio	23

To avoid scale effects, the absolute values of the original variables were swapped into rates, proportions, and location quotients. To ensure that the linear regression's normality assumption was met, data transformation [64, 65] was applied to both epidemiological information and determinant factors using the square root transformation, a common nonlinear fix used in epidemiological data analysis [66, 67].

### GIS-multicriteria susceptibility analysis

The approach proposed in this paper to derive a territorial differentiation of susceptibility to COVID-19 infection used thresholds. Following a multicriteria decision analysis, we assumed susceptibility conceptually as the definition of Porta [34] and methodologically as the likelihood of confirmed cases occurring in relation to the determinants, similar to Sarkar [16].

Other studies of susceptibility or vulnerability analysis to COVID-19 in GIS have favoured the use of multicriteria analysis based on the analytic hierarchy process [16, 68, 69]. In these knowledge-based approaches, there is a subjective influence on the relative importance of factors. In contrast, data-driven approaches based on multivariate models enable parametrizations that are based on the sensitive analysis of factors without the impact of subjectivity [70].

#### Identifying determinants of infection

The identification of determinant factors explaining the incidence patterns of COVID-19 was based on an MLR. For this purpose, the epidemiological data and the 51 potential determinants (Table 2) were considered as follows:

$$Y_{i} = \beta_{0} + \beta_{1} X_{i1 } + \beta_{2} X_{i2} + \cdots + \beta_{p} X_{ip} + \varepsilon_{i}$$

where $Y_{i}$ represents the estimated number of COVID-19 cases for the period $i$, $\beta_{0}$ is the intercept of the regression line, $X_{i}$ are the explanatory factors, $\beta_{p}$ are the coefficients for each variable and $\varepsilon_{i}$ is the mode’s error term. A stepwise algorithm was used to ensure the selection of significant independent variables. Stepping method criteria used a p-value with an entry value of 0.05 and 0.1 for removal.

#### Threshold identification

After reducing the initial set of variables and identifying the most relevant ones to explain the spatial dynamics of COVID-19, followed the stage of inventorying the existence of thresholds as change points in the relationship between determinants and disease incidence.

In this regard, we used a CPA executed with the R package ‘bcp’ [71] based on the work of Barry and Hartigan [72, 73]. In statistical analysis, CPA or step detection methods attempt to identify the moments at which the probability distribution of a stochastic process or time series changes, which have been used in epidemiological studies [74]. In this case, the probability distribution was not a time series, but the values of an independent variable in ascending order for each municipality. The link between the incidence of COVID-19 and each determinant was modelled in a bivariate approach using this method. In the specific case of the package used, a Bayesian and offline method, the abrupt changes in the posterior mean of COVID-19 incidence in relation to the determinants of infection were evaluated obtaining the a posteriori probability of change points.

#### Weighted linear combination

The results of MLR and CPA fed the WLC using 3 determinants to create the susceptibility index. The relative importance of the determinants in the outcome was calculated based on the number of periods in which they were significant in the MLR. As a result, a variable with a greater number of significant associations contributed more to the susceptibility index than one with a lower frequency of significance. With this data-driven methodology, with reduced human parameterization compared to other strategies (e.g., analytic hierarchy process), mainland Portugal was classified by susceptibility to COVID-19 infection at the municipal scale.

#### Validation

The validation of the susceptibility index was performed by calculating the area under the curve (AUC). Success rate curves were constructed for the first three waves by using the modelling data. In addition, we determined prediction rate curves for the peaks of the 4th and 5th waves that are the validation set, i.e., epidemiological data unknown to the model.

The accuracy of the classification was measured by the AUC for all the periods considered as:

$$AUC_{i} = \frac{a}{{\left( {a + b} \right)}}$$

where $AUC_{i}$ is the area under the curve for the period $i$, a is the area between the 45-degree line and the success or prediction curve and b is the area above the curve. A higher value represents a curve that with a lower cumulative percentage of the study area better captures the cumulative cases, while a lower index means higher difficulty in separability.

## Results

The results indicate that the factors examined accurately predicted the spatiotemporal dynamics of COVID-19, albeit with varying importance through time. The susceptibility analysis methodology, which combined classical and Bayesian techniques, classified municipalities according to their susceptibility to COVID-19 infection. Clusters of greater infection susceptibility were identified based on economic, sociodemographic, and mobility characteristics. In summary, the approach adopted supported the hypothesis that NPI should be specifically tailored to local geographical contexts.

### Determinants

The MLR highlighted that COVID-19 diffusion is a multifactorial phenomenon with associations varying across time. From the 51 variables for six moments, 19 were identified as statistically significant (Table 3). The number of significant factors for each moment of incidence ranged from 6 to 11, with a mean of 9. The importance of these variables, in terms of regression coefficients and statistical significance, had variability depending on the incidence period. We identified the importance of factors related to the heterogeneous occupation of the territory (population density, average family size, students enrolled of various levels), economic (income, concentrations of employment in sectors where face-to-face work is indispensable, such as textile industry and storage and auxiliary transport activities) and mobility (use of public transport, average duration of commuting by public transport, inter-municipal and interparish commuting). On the contrary, population age did not turn out to be a key factor although several indicators associated with school enrollment and employment (active population proxies) were significant.

**Table 3:** Regression model’s standardized coefficients with 95% confidence interval and variance inflation factor (VIF)

	Variables	First wave		Second wave		Third wave	
		Start	Peak	Start	Peak	Start	Peak
Sociodemografic	Population density	0.580 (0.501–0.659) VIF = 1.450	0.541 (0.458–0.624) VIF = 1.544	0.498 (0.400–0.597) VIF = 2.653	0.487 (0.402–0.573) VIF = 2.486	0.349 (0.248–0.450) VIF = 2.930	0.393 (0.299–0.487) VIF = 2.511
	Average household size	0.170 (0.098–0.242) VIF = 1.198	0.186 (0.107–0.266) VIF = 1.416)	0.268 (0.196–0.340) VIF = 1.424	0.352 (0.280–0.424) VIF = 1.764		
	Urbanization rate		0.128 (0.043–0.212) VIF = 1.579			0.209 (0.128–0.290) VIF = 1.885	0.133 (0.051–0.214) VIF = 1.864
	Students enrolled pre-school			− 0.144 (− 0.227 to − 0.060) VIF = 1.918			
	Students enrolled 2nd cycle	− 0.133 (− 0.213 to − 0.052) VIF = 1.487	− 0.164 (− 0.248 to − 0.080) VIF = 1.571		− 0.236 (− 0.314 to − 0.159) VIF = 2.054	− 0.144 (− 0.239 to − 0.048) VIF = 2.616	
	Students enrolled in higher education	0.254 (0.181–0.328) VIF = 1.239)		0.122 (0.045–0.200) VIF = 1.642	0.081 (0.005–0.157) VIF = 1.945		
Age	Population aged 0–15					0.227 (0.119–0.336) VIF = 3.355	
Mobility	Population commuting by public transport			0.090 (0.017–0.163) VIF = 1.467		0.130 (0.047–0.213) VIF = 1.985	0.209 (0.130–0.289) VIF = 1.799
	Population working/studying outside parish		0.178 (0.105–0.251) VIF = 1.199		0.148 (0.074–0.222) VIF = 1.867	0.251 (0.176–0.326) VIF = 1.622	0.198 (0.133–0.263) VIF = 1.207
	Population working/studying outside municipality			− 0.137 (− 0.218 to 0.057) VIF = 1.776	− 0.174 (− 0.260 to − 0.087) VIF = 2.562		
	Average time duration of commuting				0.154 (0.076–0.231) VIF = 2.043	0.029 (-0.055–0.113) VIF = 2.025	
Economic	Declared income					0.168 (0.069–0.267) VIF = 2.819	0.246 (0.151–0.341) VIF = 2.548
	Housing expenses				0.105 (0.036–0.174) VIF = 1.606	0.104 (0.030–0.179) VIF = 1.583	0.141 (0.070–0.211) VIF = 1.423
	Owner occupied housing			− 0.277 (− 0.368 to − 0.186) VIF = 2.282	− 0.203 (− 0.286 to − 0.119) VIF = 2.377		
	LQ textile industry		0.118 (0.044–0.192) VIF = 1.210		0.142 (0.081–0.203) VIF = 1.271	0.072 (0.008–0.136) VIF = 1.192	
	LQ vehicle trade and repair					0.080 (0.016–0.143) VIF = 1.157	
	LQ storage and auxiliary transport activities	0.154 (0.080–0.228) VIF = 1.269	0.165 (0.088–0.242) VIF = 1.314	0.170 (0.100–0.239) VIF = 1.321	0.138 (0.072–0.203) VIF = 1.471		0.081 (0.007–0.155) VIF = 1.553
	LQ electrical equipment manufacturing	0.172 (0.103–0.241) VIF = 1.108	0.130 (0.060–0.200) VIF = 1.101				
	LQ hospitality and restaurants			− 0.088 (− 0.157 to − 0.019) VIF = 1.312			

Three factors (Table 4) stood out by the number of significant moments and the relative weight of their regression coefficients:

- population density (Pdens)—sociodemographic dimension;
- proportion of population working outside the parish of residence (PWOparish)—mobility dimension;
- location quotient of employment in storage and auxiliary transport activities (LQstotrans)—economic dimension.

**Table 4:** Adjusted R^2^ comparing models with all variables versus the 3 most significant

Model	First wave		Second wave		Third wave	
	Start	Peak	Start	Peak	Start	Peak
Stepwise variables (number)	0.692 (6)	0.679 (8)	0.741 (9)	0.790 (11)	0.751 (11)	0.748 (7)
Pdens + PWOparish + LQstotrans	0.617	0.600	0.646	0.656	0.669	0.680
Difference	− 0.075	− 0.079	− 0.095	− 0.134	− 0.082	− 0.068

These three variables demonstrated a positive significant relationship with the number of new cases, e.g., the higher the variable value, the more cases the municipality tends to have and accounted for more than 60% of the variation explained by the stepwise models. An examination of the factors by dimensions reveal that Pdens was the only one with significance in all moments under study and had the highest regression coefficients. LQstotrans was the most relevant in the economic dimension and PWOparish had more frequent associations in the mobility group. The loss of explanation when eliminating the remaining variables is minimal and considering their importance these three indicators were the ones selected for CPA and later to determine the susceptibility index. The difference in the number of significant variables between the start and peak of the waves did not present a link.

### Thresholds

**Fig. 3:**
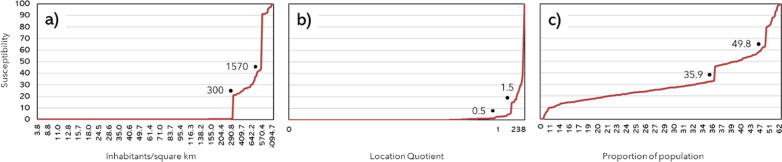
Most relevant changing points between incidence and factors: **a** Pdens; **b** LQstotrans; **c** PWOparish

The bivariate Bayesian CPA identified the posterior probability of changing points. Multiple probable points of changing relationships have been identified between incidence and determinants. To decrease the number of changes a minimum probability threshold of 0.7 was defined to assume the existence of a change in the series since this is a reference value in statistics. The trends were segmented to generalize thresholds for all the analysed periods (Fig. 3).

The Bayesian CPA highlighted that the relationship between factors and incidence depends on various changing points that trigger the posteriori mean incidence of new cases. This way, the results were suggestive of not fully linear relationships, corroborating 20 to 30% of unexplained variability of the MLR models.

Thus, the susceptibility to infection associated with each determinant is based on varying gradients that show that the influence of a determinant on the propensity to infect is not directly proportional to its value. For example, Pdens is practically irrelevant until 300 inhabitants per km^2^, while in the case of PWOparish, although non-linear, it is closer to a trajectory that could be partitioned into mulitple linear segments.

The combination of these three indicators by a WLC allowed the calculation of the susceptibility index. Also, at this stage the weight associated with each determinant resulted from the available information without subjective influence, and its importance was defined based on the proportion of the number of periods in which the respective variables demonstrated an association with the target (see Table 3). Thereby, the Pdens assumed an importance of 40%, the LQstotrans of 33% and the PWOparish of 27%.

### Spatial susceptibility index

**Fig. 4:**
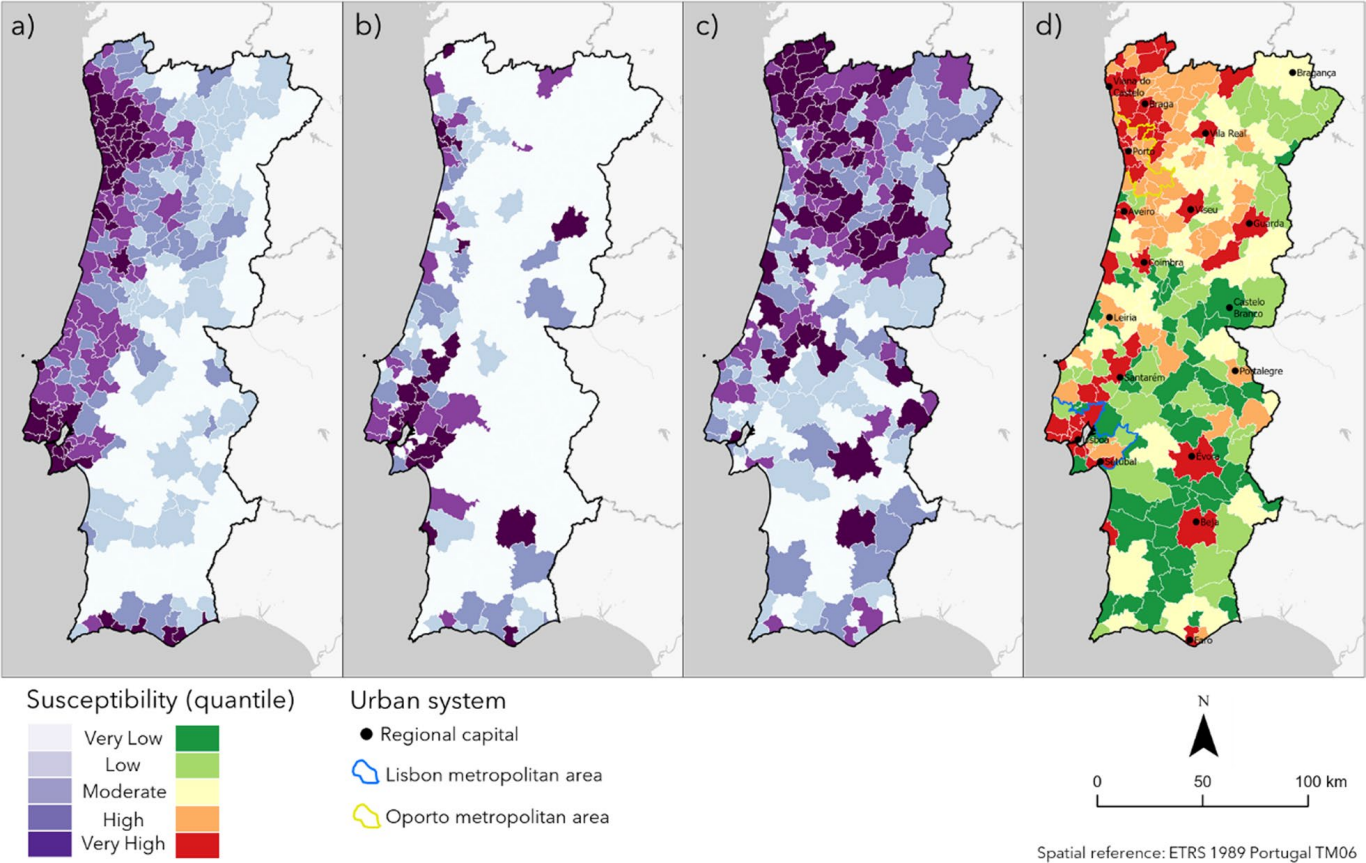
Susceptibility to COVID-19 infection in mainland Portugal: **a** Pdens; **b** LQstotrans; **c** PWOparish; **d** final susceptibility index

The spatial patterns of the susceptibility associated with each factor revealed contrasting and heterogeneous patterns, even though some municipalities were classified similarly (Fig. 4). This is reflected in the patterns of the final susceptibility index that visually replicates the influence of population density with a higher susceptibility in the metropolitan areas of Lisbon and Oporto and important urban systems such as the regional capitals. The Algarve coast in the South, albeit one of the most populous and economically dynamic regions, shows low susceptibility because LQstotrans and PWOparish have low expression in this region. This is not surprising since in the first three waves the Algarve region registered low numbers of COVID-19 infections.

The distribution of the susceptibility classes suggests the existence of specific geographic contexts influenced by the considered dimensions: sociodemographic, economic and mobility. It is also evident the influence of communication axes and the spatial dependence of the classes, i.e., the proximity, in terms of geographical distance between municipalities, seems to be relevant in terms of susceptibility. This fact is particularly evident in the case of the Northwest, where the Oporto metropolitan area demonstrated a gradient of diminishing susceptibility with increasing distance from Oporto city, but which is “inflated” by the closest regional capitals, such as Viana do Castelo or Braga. Also in the interior, the case of Guarda or Viseu is representative of this phenomenon, with adjacent municipalities classified with high susceptibility.

**Fig. 5:**
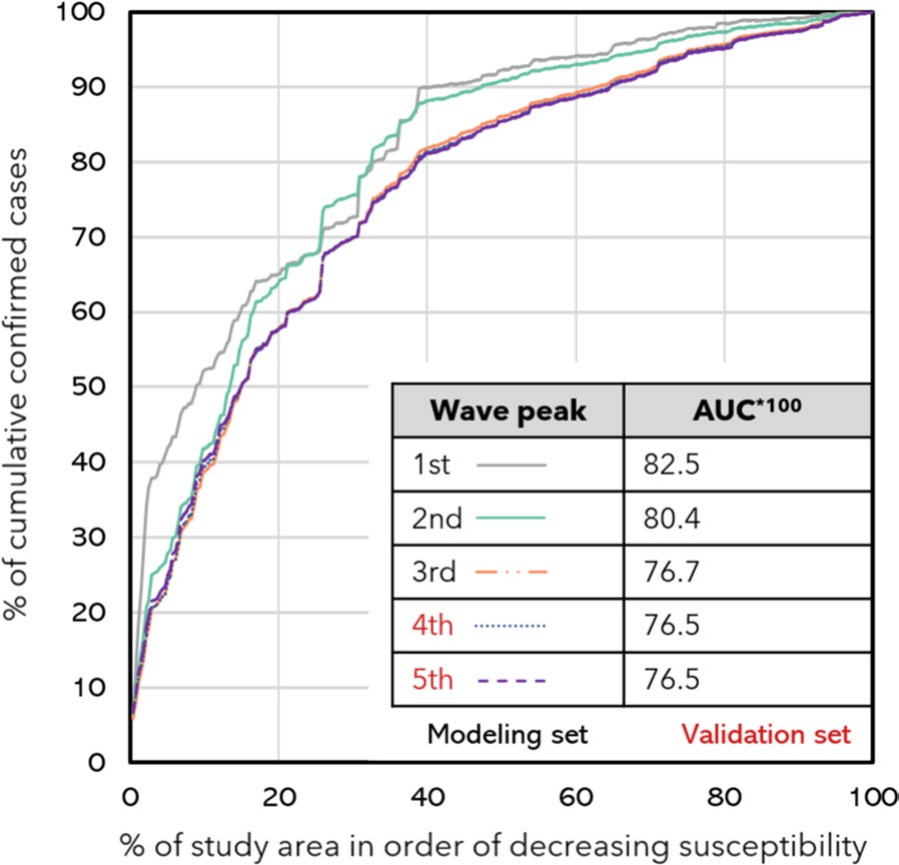
Susceptibility classes and the monthly incidence of COVID-19 during the first year of the disease in Portugal

In terms of accuracy, success and prediction rate curves revealed that 40% of the municipalities (Very High and High susceptibility) explained between 80 and 90% of the new cases of the disease (Fig. 5). The AUC were significant with values above 0.75 which is a reference for good discrimination. The validation implies that the threshold-based modelling process had significance in determining the areas with a greater propensity to register cases of COVID-19.

**Fig. 6:**
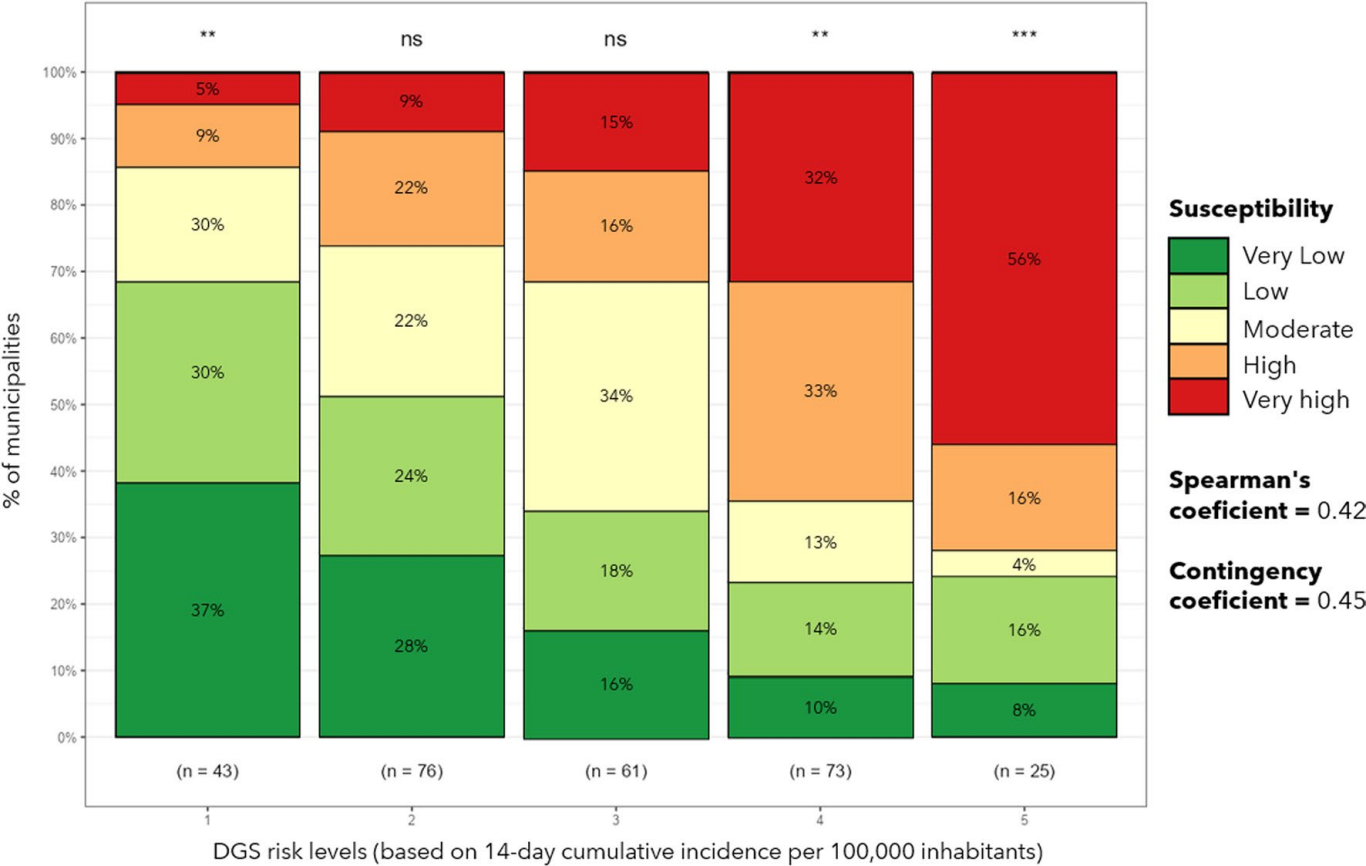
Susceptibility classes and DGS risk levels for the peak of the third wave

Comparing the susceptibility index with COVID-19 DGS risk classification (14-day incidence rate per 100,000 inhabitants) for the third wave demonstrated why integrating susceptibility and epidemiological monitoring is relevant for NPI management (Fig. 6). The comparison demonstrated little correspondence between the restrictiveness of the NPI and the susceptibility index, resulting in low contingency and Spearman coefficients.

Most of the municipalities in the highest risk level had very high susceptibility however, almost 30% had only very low to moderate. Considering the highest three levels (each had different sets of NPI with growing restrictiveness) seem to have existed overly rigid measures for several geographical contexts whose socio-territorial characteristics were not determinants of COVID-19 spread. Thus, these locations had NPI that overestimated the propensity for transmission. Also, in the first and second levels (alert levels without specific interventions) some municipalities with high and very high susceptibility stood out, presumably indicating an underestimation of outbreak potential.

Overall we can say that the correlation was low and that a disagreement between the severity of restrictions and the actual propensity for transmission was found. Therefore, the susceptibility index can be a viable instrument to support epidemiological containment policies preventing future uncontrolled transmission by imposing stricter restrictions in more susceptible areas.

## Discussion

This study identified COVID-19 infection determinants and mapped the susceptibility using a data-driven threshold approach based on only three variables, with the hypothesis that containment measures should consider not only epidemiological indicators but also the true propensity to transmission dynamics by taking geographical contexts into consideration. The results support a multi-cause aetiology for COVID-19 transmission dynamics patterns and the spatial susceptibility index highlights peculiar situations in which public health authorities may need tailored interventions.

### Specificities of the methodology

In methodological terms, some distinctive features can be mentioned. Considering that COVID-19 is often asymptomatic and under-reported [75], leading to difficulties in the identification by epidemiological monitoring and surveillance systems [76], the use of the Bayesian CPA method is justified. The uncertainty in the modelling process regarding the data itself was addressed by using posterior probabilities to define the thresholds. Furthermore, according to Nazia et al. [23] the majority of COVID-19 spatiotemporal analyses used frequentist methods, with only a minority embracing Bayesian approaches. In this sense, by combining a frequentist regression with a Bayesian method to infer transition points, the present study distances itself from more classical approaches and uses an uncommon method in susceptibility analysis. Moreover, the same authors mention the prevalence of studies addressing regional scales. However, our research focused on a local analysis that took advantage of a finer scale to improve the prediction of transmission prone municipalities.

### Summary of results

The time-varying relationships of factors identified by the MLR introduce uncertainty for an effective quantification of their real contribution. This was already identified in other studies [8, 77] and forces researchers to analyse longer periods to accurately identify and quantify the factors explaining COVID-19 diffusion. Nevertheless, the analysis of this study for a period of 1 year allowed us to unequivocally identify the importance of determinants related to the heterogeneous occupation of the territory. Urban population distribution and density, as well as household size, have a strong association with the spatiotemporal dynamics of COVID-19. Aside from a more structural view of population distribution, employment concentrations associated with regional employment specialisation and agglomeration patterns with strong interaction dynamics at regional, national, and international scales (e.g., [78]), have been linked to infection diffusion. Still, on the economic side, it is worth mentioning indicators such as income, expenses related to housing and the proportion of owner occupied dwellings. At the study’s scale, these results cannot be interpreted as indicative of socio-spatial inequalities as infection-predisposing factors, but as proxies of the most populous municipalities (because the standardized coefficients were positive) and, therefore, with more active epidemiological dynamics. Although the variables explicitly related to the age dimension had little association with the dependent variables, indicators related to employment and school enrollment were significant. This suggests that the active population was an important agent of transmission at certain times, specifically at the beginning of waves, emphasising the importance of implementing NPI associated with teleworking and mobility restrictions [48] to prevent disease transmission. It is also known that population mobility patterns are an unequivocal driver of infectious disease transmission [79] and although the data used was somewhat outdated, it showed how commuting had important links with COVID-19 transmission.

Since the MLR model’s explanatory power, albeit significant, did not exceed 70 to 80% of the variation of the dependent variable, the relationships between incidence and their explanatory factors were not completely linear. This is due to residual heteroscedasticity, which can be indicative of the need to incorporate other factors. For example, a behavioural dimension, such as adherence to NPI, mask use, containment and exposure reduction practices [80], is of extreme importance in such a study [28] but was not considered. Moreover, the existence of multiple thresholds in variables’ relationships demonstrated the importance of territorial specificities, explaining the inability of linear models to accommodate all the variations in the number of cases.

Combining the results from the MLR and a CPA, mainland Portugal municipalities were classified by their susceptibility to COVID-19 infection. Despite the complexity of infectious diseases, good model accuracy was achieved with only three variables (Pdens, LQstotrans and PWOparish). The heterogeneous geography of the index derives from the fact that the distribution of the determinants is uneven and anisotropic. The susceptibility spatial patterns resemble the distribution of confirmed cases in a trend that is “coastlised” along the most densely populated coastal areas, polarized around the country’s two metropolitan areas—Lisbon and Porto—and anchored in mainland regional capitals. The success and prediction curves followed a power distribution since most cases occurred with a high concentration in a small number of municipalities (e.g., metropolitan areas). The power distribution loses strength from the first wave—when the distribution of cases was more evident on the coast—to the subsequent waves when the infection spread to all municipalities.

The importance of economic, socio-demographic, and mobility determinants reinforces the conclusion of previous studies [8, 44, 46] for Portugal, even though the present study focused on a longer period. Municipalities with higher incidence rates coincided with the highest susceptibility classes at the peak of the third wave. However, a relevant number of outliers was identified in all the risk levels proving the that the rigidity of the restrictions was not always adequate considering the propensity to infection based on the spatial determinants conducive to COVID-19 infection. In this sense, the relevance of integrating epidemiological monitoring with susceptibility emerges as a relevant proposal in the domain of the management of NPI in Portuguese territory.

### The implications of susceptibility and future developments

The major contribution of this work is the development of a municipal susceptibility index for spatial decision-making in managing containment policies. The territorial units with higher susceptibility—most of which have spatial proximity—need to have more restrictive NPI, compared to those with lower, or adopted ahead of time to avoid severe outbreaks, due to the spatial conjugation of socio-territorial specificities that enhance transmission. The ability to adjust measures to contain the infection based on its propensity to spread is particularly important since according to Jain et al*.* [81] controlling an outbreak at the grassroots level has profound repercussions for the nationwide control of transmission chains. Furthermore, municipalities with higher susceptibility were geographically close to similar classes. As Duarte et al*.* [82] have assessed neighbouring municipalities tended to share similar behaviour because local effects justify spatial dependence in COVID-19 diffusion, confirmed in Portugal [43, 44], and which our modelling process did not account for. This is not unusual, since one of the most common processes of infectious disease spatial diffusion—contagious diffusion—is based essentially on spatial contiguity [38, 83] and which was boosted by mobility movements between municipalities. Considering this information, the geographical character of COVID-19 transmission is reinforced, strengthening the need for differentiated measures according to local contexts, e.g., spatial-based containment measures should also consider geographic properties such as proximity and contiguity (to areas of higher susceptibility).

The proposed index appears adequate for customized NPI, avoiding harsh approaches where it has no benefits and soft in contexts of rapid diffusion. Knowing also the potential negative consequences associated with NPI and long lockdown periods [84], it is important to adapt strategies to the contexts in which they fall. Despite the satisfactory results, further work is needed for a more robust spatial index considering a second order of factors and incorporating spatial dependence. Alternative approaches for a broader classification could be the use of additional epidemiological indicators such as persons hospitalized and the positivity of testing rate. Also, the use of “near real-time” mobility data, such as Google’s Community Mobility Reports [85], is relevant to forecast future cases [48] which can allow for a time-dynamic susceptibility classification. It should also be noted that infection patterns have changed with the progression of the disease, either by vaccination and/or disease variants [86], therefore identifying factors may require updating, which has direct implications for susceptibility maps.

Moreover, in light of the non-linear parameters evidenced by the CPA, it is relevant to evaluate whether the patterns of COVID-19 diffusion are indeed non-linear, or whether this non-linearity results from spatially varying processes [87]. Based on this evaluation, it may be appropriate to test dummy variables as proxies for certain territorial configurations (e.g., municipalities of metropolitan areas) and use spatial regressions or non-linear models.

The implications of the results are relevant in the context of prevention and for public health policies evaluation, something not always straightforward during the pandemic contributing to improved containment policies.

### Limitations

In methodological terms, a lack of information on some important factors may have hindered the development of an improved index. Also, DGS COVID-19 data has several known flaws [3], both in the allocation of cases to territorial units and temporal distribution, as well as loss of synchronization over time. It is unknown to what extent some quality problems with this data, which cannot be overcome, could have caused biased results. In addition, there were some periods of higher incidence, namely severe outbreaks that have no known direct explanation by the determinants [27], as occurred in migrant communities working in agricultural areas and residing in conditions of overcrowding and insalubrity [88]. The susceptibility index cannot explain these situations since they are the outcome of accidental outbreaks under very specific conditions for which there is no available explanatory data. The static character of the independent variables, and their outdated condition, were also an obstacle to better adjustments since numerous high-magnitude changes have happened, such as variations in mobility patterns [48]. Finally, given that the results stem from aggregated units, there is the influence of modifiable area unit problems as well as ecological fallacy [89, 90] which means that the results should not be extrapolated to individual-level.

## Conclusions

The present study demonstrated how the integration of susceptibility to COVID-19 infection, based on the distribution of the known determinants and their effects, is relevant for policy guidance and containment strategies in specific geographic contexts using Portugal as a case study.

The results shed new light on how knowledge of the distribution of factors explaining transmission is crucial to identify locations where higher incidence is expected by the conjugation of sociodemographic, economic and mobility characteristics. By using factors with proven explanatory power in COVID-19 diffusion in mainland Portugal, we proposed a susceptibility index to implement spatial-based NPI.

Conclusions can be summarised in three points:

1. The MLR results showed that the importance of determinants to COVID-19 infection had time-varying contributions, although there are three with consistent relationships over time: population density, inter-parish commuting and employment in storage and transport auxiliary activities.
2. The bivariate probabilistic CPA revealed a non-linear nature of the relationships between infection determinants and observed incidence, allowing the identification of thresholds as transition points in changing trends.
3. Comparing the susceptibility classes with the risk levels for NPI evidenced low correlation, suggesting the need for considering susceptibility as a criterion together with epidemiological monitoring.

The findings prove that the portuguese NPI strategy was poorly adjusted to the reality of the propensity to COVID-19 spread. In summary, the results lay the groundwork for future models that intersect incidence rate with the susceptibility to infection for NPI management, advocating the need for greater incorporation of spatial variables in epidemiological containment policies. It is also noteworthy that, unlike previous studies, this one followed a data-driven approach based on thresholds, reducing subjectivity when compared to previous studies using multicriteria analysis. The approach can be extended to other regions of the world for the current or future epidemic(s).

## Data Availability

Not applicable.
